# The Pancreas and Known Factors of Acute Pancreatitis

**DOI:** 10.3390/jcm11195565

**Published:** 2022-09-22

**Authors:** Julia Walkowska, Nicol Zielinska, Piotr Karauda, R. Shane Tubbs, Konrad Kurtys, Łukasz Olewnik

**Affiliations:** 1 Department of Anatomical Dissection and Donation, Medical University of Lodz, 90-419 Lodz, Poland; 2 Department of Neurosurgery, Tulane University School of Medicine, New Orleans, LA 70112, USA; 3 Department of Neurosurgery and Ochsner Neuroscience Institute, Ochsner Health System, New Orleans, LA 70121, USA; 4 Department of Anatomical Sciences, St. George’s University, St. George’s P.O. Box 7, Grenada; 5 Department of Neurology, Tulane University School of Medicine, New Orleans, LA 70112, USA; 6 Department of Structural and Cellular Biology, Tulane University School of Medicine, New Orleans, LA 70112, USA; 7 Department of Surgery, Tulane University School of Medicine, New Orleans, LA 70112, USA

**Keywords:** pancreas, surgery, risk factors, pancreatitis

## Abstract

Pancreatitis is regarded by clinicians as one of the most complicated and clinically challenging of all disorders affecting the abdomen. It is classified on the basis of clinical, morphological, and histological criteria. Causes of acute pancreatitis can easily be identified in 75–85% of patients. The main causes of acute, recurrent acute, and chronic pancreatitis are gallstone migration and alcohol abuse. Other causes are uncommon, controversial, or unexplained. For instance, cofactors of all forms of pancreatitis are pancreas divisum and hypertriglyceridemia. Another factor that should be considered is a complication of endoscopic retrograde cholangiopancreatography: post-endoscopic retrograde cholangiopancreatography acute pancreatitis. The aim of this study is to present the known risk factors for acute pancreatitis, beginning with an account of the morphology, physiology, and development of the pancreas.

## 1. Introduction

The pancreas is a secondary retroperitoneal organ located in the upper part of the abdomen. Macroscopically, it has conventionally been divided into three main parts: the head, the body, and the tail [1]. Anatomically, some structures such as the uncinate process are commonly regarded as parts of the head, which is located below the superior mesenteric artery and vein, and the neck, situated above those vessels [2,3]. The head is surrounded by a loop of the duodenum. The body, extending almost perpendicular to the median plane, is located below the stomach. It crosses the superior mesenteric artery and vein, the abdominal aorta, the inferior vena cava, and the hepatic portal vein. Finally, the tail approaches the hilum of spleen.

The pancreas is a key organ in overall body homeostasis; it is responsible for regulating macronutrient digestion and releasing hormones that control digestive processes and the blood glucose level. It has two distinct components: the exocrine pancreas and the endocrine islets [4,5]. Anatomical variations of the pancreas and pancreatic ducts are uncommon. The structure of the pancreatic ducts is normal in 94.3% of cases; the remaining 5.7% show many different variations, many of which remain asymptomatic and undetected until adulthood. They are frequently detected as incidental findings on clinical imaging [1]. Distinct diseases affect the exocrine and endocrine pancreas. They can affect physiological functions or arise from pancreatic dysfunction. Those concerning the exocrine pancreas are acute and chronic pancreatitis [6]; pancreatic cancers [7], most of which are ductal carcinomas; cystic fibrosis [8]; and pancreatic insufficiency leading to malabsorption syndrome [9]. Diabetes [10,11] and rare pancreatic neuroendocrine tumors arise from the endocrine islets [5,12].

A range of causes, symptoms, and outcomes are associated with pancreatitis. In this article we argue that familiarization with the morphology, physiology, and biochemistry of the organ facilitates a more accurate understanding of the pathomechanisms of disease, which has a positive effect on diagnostics and treatment, in turn increasing the chance of successful treatment and improvement of the patient’s condition. This review also summarizes the information published so far about the most important risk factors for acute pancreatitis (AP).

## 2. Morphology and Physiology of the Pancreas

**Figure 1:**
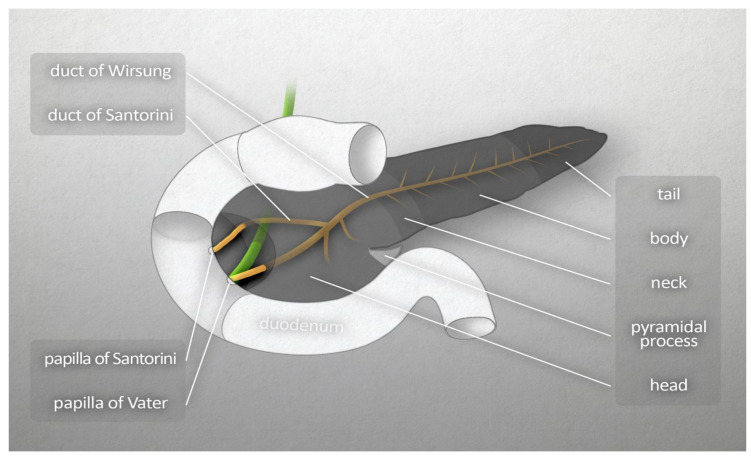
Macroscopic anatomy of the human pancreas.

On the basis of the anatomical structure and embryonic development of the pancreas, Suda et al. [2] proposed that the organ be divided into four segments: anterior head, posterior head, body, and tail. There are no clear boundaries between these segments. However, it is generally considered that the boundary between the head and the body is the left border of the superior mesenteric vein, while the boundary between the body and the tail has no anatomical determinant and is therefore taken as the midpoint of the total length of body and tail [2]. Figure 1 presents the schematic anatomy of the pancreas.

### 2.1. Vascularization

**Figure 2:**
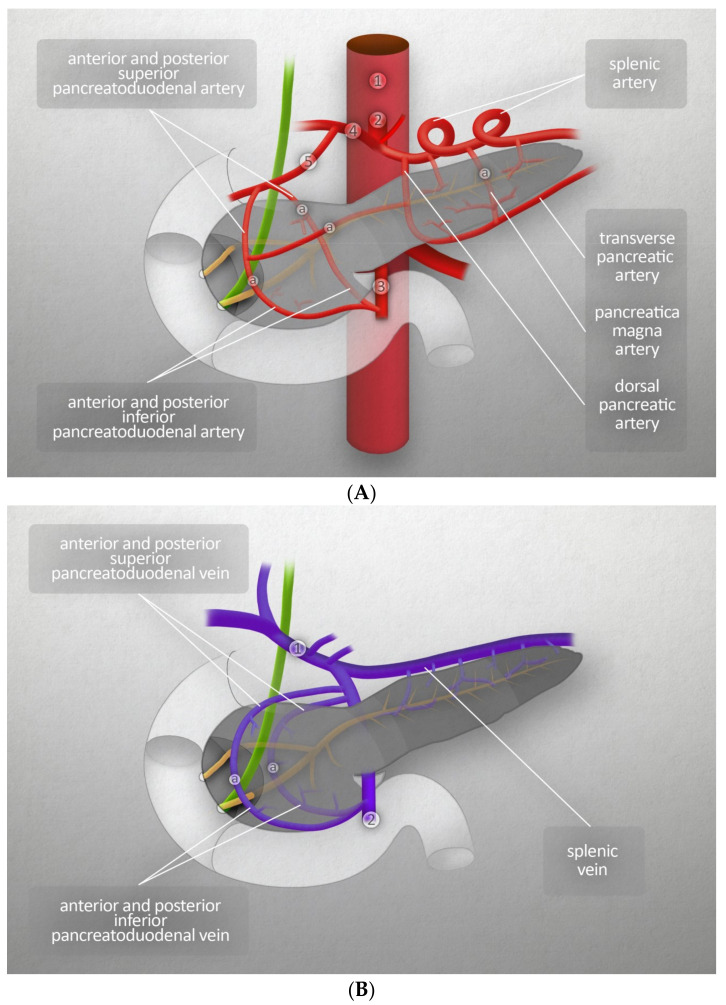
(**A**) Major blood vessels of the pancreas- arteries: (1) abdominal aorta, (2) celiac trunk, (3) superior mesenteric artery, (4) common hepatic artery, (5) gastroduodenal artery, (a) anastomotic branch. (**B**) Major blood vessels of the pancreas-veins: (1) hepatic portal vein, (2) superior mesenteric vein, (a) anastomotic branch.

The amount of blood reaching the pancreas is about 1% of the cardiac output. The arterial supply comes from two branches of the abdominal aorta: the celiac trunk and superior mesenteric artery. The head of pancreas is supplied through the anterior and posterior arcades. The anterior arcade consists of the anterior superior pancreaticoduodenal artery (PDA), an indirect branch of the celiac trunk (it stems from the gastroduodenal artery, which is a branch of the common hepatic artery, itself a branch of the celiac trunk), and the inferior superior PDA, which is a branch of the superior mesenteric artery. The posterior arcade is formed by the posterior superior PDA, the first branch of the gastroduodenal artery, and the posterior inferior PDA, which stems from the superior mesenteric artery. The body and tail of the pancreas are supplied by the pancreatic branches of splenic artery and dorsal pancreatic artery (the latter branching from the celiac trunk near its origin), the common hepatic artery, or the splenic artery. On its right side, the dorsal pancreatic artery forms anastomoses with the anterior superior PDA, while its left branches form the inferior pancreatic artery. It runs along the inferior border of the organ and often connects to the great pancreatic artery, the largest branch of the splenic artery that supplies the pancreas. Venous blood from the pancreas flows into the hepatic portal vein. The venous vascularization of the head is similar to its arterial vasculature. The venous vascularization of the body and tail is more variable than the arterial; venous blood flows mainly into many small branches of the splenic vein [3]. Figure 2A,B present major blood vessels of the pancreas. Those figures also show the vascularization of the head of the pancreas by the anterior and posterior arcades, both arterial and venous, and the origins of the branches that merge to form those anastomoses [3].

### 2.2. Innervation

The pancreas is a richly innervated organ. Sympathetic, parasympathetic, and afferent fibers enter or exit the pancreas as neurovascular stalks. The fibers follow blood vessels, beginning or ending close to capillary walls and endocrine cells. The ends of the fibers do not form classical synapses with target cells, but release neurotransmitters into the extracellular space, thereby affecting more than one cell at a time. The fibers innervating the pancreas project from the prevertebral ganglia, directly or within the mixed autonomic nerves. The majority of fibers supply the head of pancreas. It is innervated by two plexuses originating from the celiac plexus—the anterior hepatic plexus distributed along the common hepatic artery, and the posterior hepatic plexus surrounding the posterior side of the hepatic portal vein. While the tail and the body of pancreas are innervated by fibers from the celiac plexus, that accompany 2 arteries, namely the inferior pancreatic artery, and the splenic artery around which fibers create the splenic plexus. Additionally, the nerve fibers running along the inferior pancreaticoduodenal artery, projecting from the superior mesenteric ganglion, supply the uncinate process [3].

### 2.3. Lymphatic System

The lymphatic system of the pancreas can be divided into internal and external systems. The internal lymphatic system begins with intralobular lymphatic vessels, running along the smallest ducts and blood vessels in the connective tissue of the intralobular septa. Intralobular lymphatic vessels merge into interlobular lymphatic vessels, which pass through the connective tissue that separates the lobules together with interlobular ducts and blood vessels. The internal and external lymphatic systems are connected by the largest interlobular vessels, also called collecting vessels, which reach the surface of the pancreas and drain into the surface network of lymphatic vessels, the beginning of the external lymphatic system. Then, the lymph is transported to the regional lymph nodes by the larger lymphatic vessels. Seven main groups of lymphatic vessels can be distinguished, associated with corresponding groups of blood vessels. Two groups drain the left part of the body and the tail of the pancreas: the superior vessels (along the splenic blood vessels) and inferior vessels (along the transverse pancreatic artery). The remaining five groups collecting lymph from the right part of the body and the head of the pancreas are the anterosuperior, anteroinferior, posterosuperior, posteroinferior pancreaticoduodenal vessels (following the corresponding arteries), and the gastroduodenal vessels [3,13].

There are two main groups of pancreatic lymph nodes. Their names correspond to their locations and the names of the blood vessels that run beside them. The lymph nodes belonging to the first main group are located on both sides of the aorta near the splenic hilus, in the area of the liver and duodenum, and along the corresponding blood vessels. The second group is related to the abdominal aorta and its trunks [3].

Products of both the endocrine pancreas (e.g., insulin) and the exocrine pancreas (digestive enzymes) are present in the lymph leaving the organ and flowing into the thoracic duct. Insulin enters the lymph by leakage into the interstitial fluid from the pancreatic islets or right into the lymphatic vessels lying close to the interlobular islets. There is evidence implicating insufficient removal of pancreatic proteolytic enzymes and extracellular fluid from the interstitium in the etiology of pancreatitis. This leads to inflammation and subsequent injury to the interstitium and lymphatic vessels, initiating a vicious cycle. Edema is increased as lymphatic vessels are increasingly unable to drain the fluid, and further injury results. Ultimately, multiple and progressing inflammation and fibrosis of the pancreas predispose to recurrent acute and chronic pancreatitis (CP) [3,14].

### 2.4. Exocrine and Endocrine Functions

The pancreas comprises two distinct components: the exocrine pancreas and the endocrine islets. The exocrine pancreas, which constitutes most of the gland, consists of acinar and ductal cells. It generates the pancreatic fluid/juice secretion composed of water, ions, and digestive enzymes such as amylase, pancreatic lipase, and trypsinogen. This secretion is released into the pancreatic ducts, which finally merge and form the pancreatic duct and accessory pancreatic duct, and flow into the small intestine, initially the duodenum, to break down nutrients for absorption [4,5,12]. The endocrine islets comprise five different cell types, which secrete hormones directly into the blood stream. Each hormone has distinct functions. Insulin (beta cells) decreases blood glucose levels, whereas glucagon (alpha cells) has an antagonistic effect, increasing blood glucose levels. Somatostatin (delta cells) inhibits glucagon and insulin release, while pancreatic polypeptide (PP cells) regulates both the exocrine and endocrine secretion activities of the pancreas. Together, these hormones are responsible for glucose homeostasis [4].

## 3. Anatomical Variations

The variations of pancreatic ducts are uncommon. They concern 5.7% of cases. The imaging techniques used to evaluate the pancreas and pancreatic ducts are computed tomography (CT) scans of the abdomen, endoscopic retrograde cholangiopancreatography (ERCP), which is considered the gold standard, and magnetic resonance cholangiopancreatography (MRCP), a noninvasive technique without contrast agent. Anomalies include annular pancreas, ectopic pancreas, agenesis and hypoplasia of the pancreas, and accessory pancreatic lobe [15].

The anatomical variations and developmental anomalies of the pancreatic ducts include configuration variants (bifid configuration with dominant pancreatic duct (PD), dominant accessory pancreatic duct (APD) without divisum, pancreas divisum, absent APD, ansa pancreatica, and cystic dilations of terminal portions of the PD and APD, course variants (descending, vertical, sigmoid, loop-shaped and ring-shaped), duplication anomalies, and anomalous pancreaticobiliary ductal junction (APBU). The most common variants of the pancreatic ducts are a descending course (50%), and bifid configuration with a dominant pancreatic duct (60%). Less common are an absent accessory pancreatic duct (30%), a dominant accessory pancreatic duct without divisium (1%) and ansa pancreatica, in which a side branch of the pancreatic duct is connected to the accessory pancreatic duct forming a reversed-S shape. Recognition and awareness of these anomalies enable the course of ERCP and surgical procedures to be planned appropriately. At the same time, they prevent inadvertent injury to the pancreatic ducts. On the other hand, anatomical variability can be a cause of recurrent pancreatitis or of gastric outlet obstruction [1,15].

**Figure 3:**
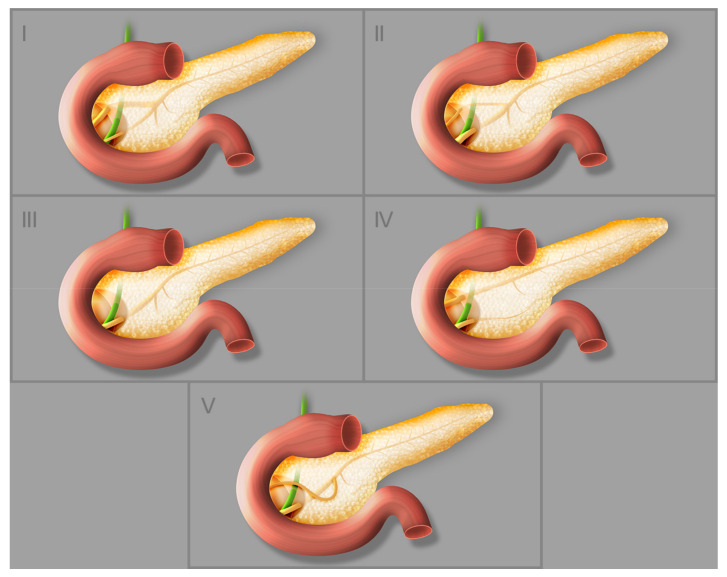
Different types of pancreatic duct configuration: (**I**) bifid configuration with pancreatic duct as the dominant duct, (**II**) bifid configuration with accessory pancreatic duct as the dominant duct, (**III**) rudimentary nondraining or absent accessory pancreatic duct, (**IV**) pancreas divisum, (**V**) ansa pancreatica.

Dimitriou et al. [1] analyzed 8260 patients and determined the incidences of the types of pancreatic ductal system presented on the Figure 3. The pancreatic ducts (types 1–3) were anatomically normal in 94.3% of cases; 4.5% had pancreas divisum and the remaining 1.2% had rare abnormalities such as ansa pancreatica (0.25%), duplication anomalies, annular pancreas, Santorini cele, anomalous pancreaticobiliary ductal junction (APBU), and undefined rare anomalies [1]. Santorini cele, a focal cystic dilatation of the terminal part of the dorsal pancreatic duct, is a rare disorder that occurs in patients with pancreatitis, mainly chronic pancreatitis. In most cases it is accompanied by pancreas divisum [16].

## 4. Embryology and Histology of the Pancreas

### 4.1. Embryology

The first signs of pancreas formation appear at gestational day 26. Initially, a dorsal bud forms, followed by the right and left abdominal buds at approximately gestational day 30. The left ventral bud gradually regresses, while the right bud starts to migrate posteriorly circa gestational day 35 and eventually merges with the dorsal bud after gut rotation at 6–7 weeks. Most of the pancreas, i.e., the anterior part of the head, the isthmus, the body, and the tail, develops from the dorsal bud; the right ventral bud forms the posterior part of the head, which completely or partially surrounds the common bile duct and includes most of the uncinate process [2,17].

### 4.2. Histology

The pancreas is surrounded by connective tissue of mesenchymal origin, which forms a fibrous capsule and gives rise to the septa that extend from the capsule into the gland and divide the parenchyma into distinct lobes and lobules. The microscopic structure of the pancreas reflects its function an endocrine and exocrine gland [3,4].

The exocrine pancreas, comprising the secretory portion and excretory ducts, accounts for 96–99% of the total volume of the organ. Each lobule consists of acini, which are clusters of pyramidal acinar cells that synthesize and secrete pancreatic juice into the lumina of the intercalated ducts. These ducts drain into intralobular ducts, which in turn drain into interlobular ducts, which have a larger diameter. These converge into the pancreatic duct (also called the duct of Wirsung) which extends throughout the length of the pancreas and together with the common bile duct empties into the duodenum. The most terminal parts of both ducts create the hepatopancreatic ampulla (also called the ampulla of Vater) which opens into the duodenum through the great duodenal papilla (major duodenal papilla). In some cases, the human pancreas has an accessory pancreatic duct (the duct of Santorini), which is the remnant of the terminal part of the main duct of the dorsal bud present during embryogenesis [3,4].

The remaining 1–4% of the total volume of the pancreas constitutes the endocrine part, the islets of Langerhans. These contain at least five types of polypeptide-hormone-secreting endocrine cells, each numbering a few to several thousand. The most numerous cells (65–80%) are beta cells, which synthesize and secrete insulin, amylin, and C-peptide. Alpha cells, accounting for 15–20% of the total number, secrete glucagon, one of the hormones antagonistic to insulin. The least common types are delta cells (3–10%) and PP cells (3–5%), which secrete somatostatin and pancreatic polypeptide, respectively. Finally, fewer than 1%, the epsilon cells, release the hormone ghrelin [3,4].

The islets receive approximately ten times more blood per unit mass than the exocrine part of the pancreas. The microvasculature of the pancreas consists of the smallest intralobular arteries: arterioles and capillaries. There is also a system of connections between capillaries called the insulo-acinar portal system: the blood from the islets is drained into efferent capillaries that form secondary capillaries, which supply the acinar cells [3].

**Figure 4:**
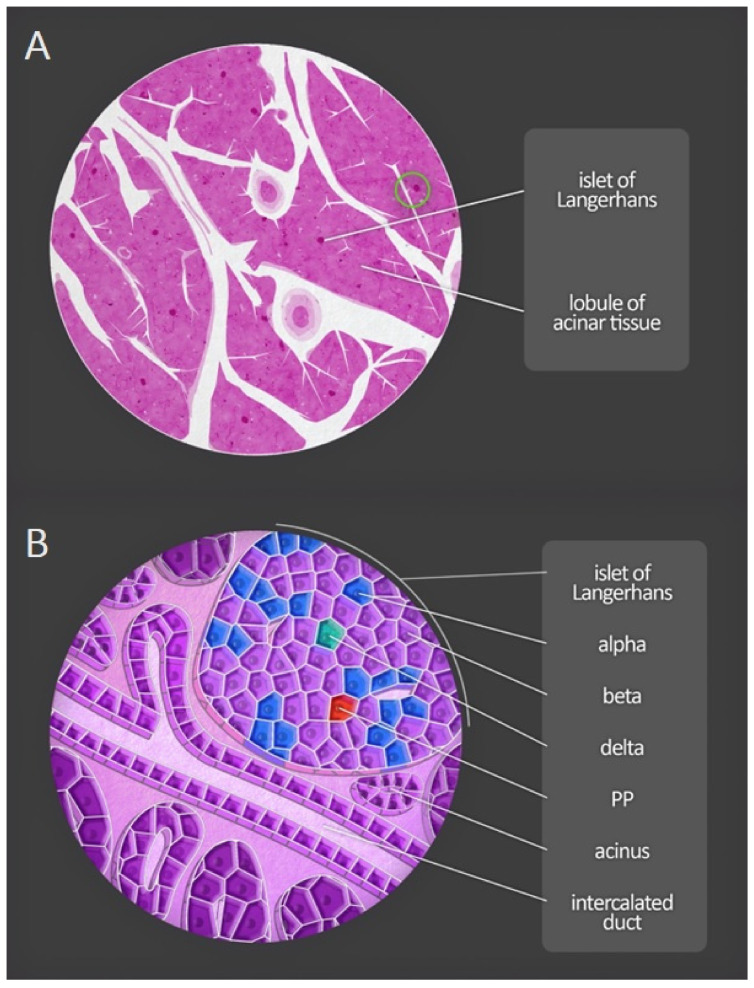
Microscopic anatomy of the human pancreas. (**A**) Magnification of a portion of the pancreas shows both lobules and pancreatic islets. (**B**) At greater magnification, acini and excretory ducts are visible. Different types of endocrine cells composing the pancreatic islets can be distinguished by immunofluorescent staining. PP—pancreatic polypeptide cells.

Figure 4 presents diagrammatic anatomy of the pancreas visible in the microscope-lobules and pancreatic islets, as well as acini and excretory ducts at greater magnification.

### 4.3. Histopathology of Acute Pancreatitis

Acute and chronic types of pancreatitis can be distinguished histopathologically. However, some samples contain features of both variants. The initial phase of AP is called edematous, but it is difficult to identify in human materials. The earliest changes demonstrated experimentally (within 3–6 h following the action of a specific injurious factor) were separation of the duct epithelial cells, edema, and disruption of cytoplasmic organelles in the acinar cells. Fat necrosis and/or necrosis of pancreatic tissues ensued (in some cases this resulted from vascular occlusion). Each of the above-mentioned changes can dominate and can be associated with hemorrhage. Two types of acute lesion can be distinguished: necrosis of the duct epithelium with periductal acute inflammation, and perilobular necrosis, perhaps caused by hypoperfusion. The changes characteristic of chronic inflammation include fibrosis, duct abnormalities, calcification, and inflammatory infiltrate. Fibrosis (perilobular and panlobular) indicates a previous inflammatory process. Stenosis, alternating dilation and segmental dilation of the ducts, is also observed. The pancreatic islets can show compensatory hypertrophy. The pathological definition of pancreatitis is unambiguous because similar changes occur in elderly patients who have not been diagnosed with pancreatic disease. In such cases, the microscopic image of the pancreas is characterized by dilatation of the ducts, fibrosis, calcium deposits and protein plugs, and metaplasia [18].

## 5. The Course of Pancreatitis

Pancreatitis is an inflammatory disease, most commonly initiated by factors such as moderate alcohol consumption and gallstones, not by infectious agents. The pathogenesis and course of the disease can be triggered or affected by interactions between genes and the environment [6,19].

Nowadays, the concept of a disease continuum has replaced the paradigm that acute, recurrent, and chronic pancreatitis are different entities. It has been shown that 30% of patients with AP go on to develop a chronic form of disease, often suffering recurrent pancreatitis during the interim [6]. Therefore, although this review is focused on AP, basic information about recurrent acute pancreatitis (RAP) and CP is necessary and draws attention to specific preventive and therapeutic measures such as lifestyle modification or surgical treatment (for instance less alcohol consumption, low-fat diet, preventive cholecystectomy) used in the management of AP.

### 5.1. Acute Pancreatitis

AP is one of the most common among The International Classification of Diseases (ICD) diagnoses for hospitalization in Europe and the USA, with an incidence of 13–45/100,000 [6,20]. It is diagnosed on the basis of two of three criteria: radiological imaging signs of pancreatitis, typical belt-like abdominal pain, and a serum lipase level three times above normal. Neither gender predominates [19]. However, an alcoholic etiology is more common in males, whereas in women the condition is often associated with biliary pancreatitis [19]. Including all etiologies, the overall risk for AP increases with age. Patients with a first attack of AP are typically in their sixth decade of life. There is also acute idiopathic pancreatitis, the incidence of which increases with age and reaches a plateau after 65 years [19]. AP can be a mild, self-limiting disease that requires only supportive measures, but can also turn into a severe disorder with life-threatening complications such as respiratory and cardiovascular insufficiency or kidney failure [19,21].

There are five subtypes of AP, differing in mortality rates: mild AP, moderately severe AP, severe AP, interstitial edematous AP, and necrotizing AP [19]. For severe, necrotizing AP the mortality rate reaches 25%, while mild, edematous AP has a mortality of only 1%. Among all patients suffering AP, 20–30% experience recurrent pancreatitis attacks and 10% of these develop CP [22,23,24].

As mentioned below, CP may be manifested by diabetes mellitus. Interestingly, the study by Chen et al. [25] presented that on the basis of genetic predisposition, the immune response during AP results in immune damage and severe destruction of β-cells. Therefore, AP may be a pathogenic factor of fulminant type 1 diabetes mellitus (FT1DM), which is characterized by a rapid loss of β-cell functions, negative antibody to islet cells, ketoacidosis, and elevated pancreatic enzymes [26].

Clinical data confirm that AP, RAP, and CP form a disease continuum. According to the necrosis–fibrosis sequence hypothesis, recurrent attacks of AP lead to CP, which is caused by the replacement and healing of areas of necrosis by fibrotic tissue [6].

### 5.2. Recurrent Acute Pancreatitis

RAP, described as the occurrence of at least two separate evidenced episodes of AP with asymptomatic intervals and morphological signs of a normal gland, is an intermediary stage in the development of CP. RAP is diagnosed retrospectively after the second episode. Often, the symptoms of CP are recognized after the first attack of pancreatitis or during a relapse, suggesting that the distinction between RAP and CP is fluid. Patients with RAP are younger than those with first-attack AP, typically in their fourth or fifth decade of life, with a male predominance. This difference is probably related to a higher risk of recurrence in younger patients and a narrower spectrum of etiologies (i.e., fewer gallstones, more intensive drinking behavior). Younger patients show a higher risk of RAP and CP through continued alcohol consumption [6,23,24].

### 5.3. Chronic Pancreatitis

The prevalence of CP totals 13–52/100,000 [27]. CP is described as a continuous, irreversible inflammatory degeneration of the pancreas. This leads to chronic mid-epigastric abdominal pain with radiation to the back associated with the loss and fibrosis of the acinar cells and finally to insufficiency of the endocrine and exocrine pancreas. There are irreversible alterations in the histology and anatomy of the pancreas, typically duct changes, calcium deposits, pseudocysts, or fibrosis. Other manifestations are a gradual loss of exocrine function, leading to malabsorption and steatorrhea and consequently to weight loss and endocrine insufficiency, causing glucose intolerance and eventually type 3c diabetes mellitus (T3cDM). Unfortunately, these patients also suffer recurrent pain attacks. T3cDM is a metabolic dysfunction secondary to the injury to the pancreas. Like type 1 diabetes mellitus it is insulin-dependent, but in T3cDM glucagon production is also impaired because the destruction of pancreatic tissue during the inflammatory process is non-selective. CP shows a male predominance and is usually diagnosed between the fifth and sixth decades of life. It is commonly associated with alcohol consumption. However, the peak incidence of alcohol-induced AP occurs a decade earlier than alcohol-related CP. A rare form, hereditary pancreatitis, is consequent on autosomal dominant mutations in the gene for the cationic trypsinogen (PRSS1). The disorder starts in childhood and progresses to CP during the second or third decade of life, advancing through variable clinical recurrences [6]. Owing to infections, malnutrition, and complications from recurrent pancreatitis, the mortality rate of CP is up to 50% within 20–25 years of diagnosis. Additionally, CP is presumed to be a risk factor for pancreatic cancer [6,19].

There are also two subtypes of chronic idiopathic pancreatitis: early-onset, which occurs during the third decade of life, and late-onset, affecting patients during the sixth to seventh decades [6].

### 5.4. Clinical Aspects

Two phases of AP have been identified: early and late [28]. The revised Atlanta classification system from 2012 [19] distinguished two morphologic subtypes of AP: interstitial oedematous pancreatitis and necrotizing pancreatitis. Additionally, the classification divided AP, on the basis local or systemic complications as well as presence and duration of organ failure of into 3 subtypes: mild AP, moderately severe AP, and severe AP. Mild AP is the most frequent form that usually resolves in the first week, and is also characterized by no organ failure and no local or systemic complications. However, moderately severe AP is associated with transient organ failure resolving within 48 h, local or systemic complications without persistent organ failure, and exacerbation of co-morbid disease. Finally, patients with severe AP suffer from persistent organ failure lasting > 48 h [19,29]. The mortality is different among the subtypes of AP. For instance, severe AP leads to mortality rate reaching 25%, while mild AP includes a mortality of only 1%. Overall, 20–30% of patients suffering from AP experience recurrent pancreatitis attacks, and of these, 10% develop CP [22,23,24].

Pancreatitis can lead to local or systemic complications. Each of those has its own characteristics based on the patients’ symptoms. Acute peripancreatic fluid collections, pancreatic and peripancreatic necrosis (sterile or infected), pancreatic pseudocyst, and walled-off pancreatic necrosis (sterile or infected) are the local complications of AP, whereas insufficient function of cardiovascular, respiratory, and urinary systems are systemic complications of AP [19,21].

AP can be a mild, self-limiting disease that requires only supportive care, but also it might turn into severe disorder with life-threatening complications [19,21]. Patients who have persistent organ failure, categorized as severe AP, have the highest risk of death. Therefore, patients with severe AP should be admitted to an intensive care unit and constantly monitored. Accordingly, diagnosis and prediction of severe AP is crucial, as well as identifying the patients with high risk of developing complications [29].

Early initiation of diagnostics to determine the etiology increases the probability of stating a proper diagnosis. Additionally, it enables the implementation of appropriate treatment, methods to prevent complications, and allows measurements to be taken to prevent subsequent attacks of pancreatitis. Therefore, the etiology of AP should be determined on admission. The etiology is defined on the basis of detailed personal and family history of pancreatic disease, physical examination, laboratory serum tests, and imaging. Another point that should be taken on admission is to predict outcome of AP. It is advised to evaluate host risk factors, clinical risk, and response to initial therapy [30].

To predict the severity and mortality of AP, clinical data (including assessment of organ function) are assessed, laboratory tests (serum lipase and amylase) and imaging are performed, and severity-of-the-disease rating systems are used, such as Ranson’s criteria [31], Acute Physiology and Chronic Health Evaluation II (APACHE II) [21], Balthazar CTSI scoring (CTSI) [32], Mortele Modified CTSI scoring (M-CTSI) [33], Bedside Index of Severity in Acute Pancreatitis (BISAP) [34], Harmless Acute Pancreatitis Score (HAPS) [35,36,37], and first artificial intelligence model-EASY-APP [29,30,38].

As mentioned above, the risk of recurrence after the first attack of AP is about 20% and the percentage of patients developing CP after at least one recurrence reaches 35% [39]. It is important to remember that most patients with CP have at least one attack of AP in their medical history; however, up to one third of patients could have had subclinical episodes of AP or no history of AP at all [40].

When commonly-used and mostly non-invasive diagnostic methods such as laboratory tests, clinical symptoms and imaging techniques give results that meet the criteria for AP, the disease can be diagnosed easily and quickly, which positively affects prognosis. In contrast, a clear and unambiguous diagnosis of CP requires a pancreatic biopsy, which, like any invasive procedure, entails complications. However, it provides valuable diagnostic material showing characteristic pathological changes in the pancreatic tissue, e.g., fibrosis or calcification [19,41].

Diagnosing CP at an early stage can be challenging because it is difficult to detect the subtle histological and morphological changes. More obvious morphological changes, mainly fibrosis of the pancreatic tissue, tend to develop over months or years. Additionally, clinical symptoms such as pain and exocrine insufficiency do not always correlate with the morphological features of CP [19,41]. Therefore, waiting for advanced CP symptoms to develop can reduce the chances of effective early intervention.

As assumed, RAP is an intermediate stage in the pathogenesis of CP. In a subset of patients, it is a natural transition to CP. Diagnosis at a given point in the continuum has clinical importance in enabling preventive measures to be taken before the irreversible changes of end-stage CP are established [24].

## 6. Known Risk Factors and Pathomechanism of Acute Pancreatitis

The etiology and pathogenesis of AP have been the focus of research globally for many years. The first hypothesis about the pathomechanism was advanced by Claude Bernard in 1856 [42]: the disease could be caused by bile reflux into the pancreatic duct. In 1901, Eugene Opie [43,44] suggested that migration of a gallstone into the common bile duct triggers AP. Multiple other causes have been uncovered since then [45]. They can be easily identified in 75–85% of patients; gallstone migration obstructing the common bile duct (38%) and alcohol abuse (36%) are the most frequent causes in developed countries [46,47].

Other causes depend on combinations of circumstances and are rare or controversial. AP develops when the intracellular protective mechanisms of the pancreas that prevent trypsinogen activation or reduce trypsin activity are overwhelmed [48].

There are many hypotheses concerning the pathogenetic mechanism of AP. Important ones involve pancreatic autodigestion, the bile–pancreatic duct common pathway, gallstone migration, microcirculation disturbance, enzyme activation, pancreatic acinar cell apoptosis, necrosis, kinin and complement system activation, and excessive leukocyte activation [49]. They are all controversial because the course of AP is rapid and access to pancreatic tissue for examination during the course of pancreatitis is difficult, so an examination aimed at determining the pathogenesis of the disease is impossible. Therefore, researchers have used animal models to study the pathological process and develop targets for therapeutic intervention [49].

A question that researchers try to address is whether each cause of AP is related to a specific pathogenic process or whether different causes lead to a common pathogenic pathway. For instance, Wang et al. [49] hypothesized that acute biliary pancreatitis and other causes of AP have a common pathogenesis. In other words, a common pathogenesis underlies different forms of AP, acute biliary pancreatitis, and others. However, all views about the pathogenesis of the disease remain controversial.

Recently, the role of systemic inflammation in pancreatitis has been proved. Early protease activation and NFκB (nuclear factor kappa-light-chain-enhancer of activated B cells) activation are essential characteristics of pancreatitis, as both events occur in parallel during disease manifestation and strongly influence each other. The activation of NFκB is one of the first step during pancreatitis and occurs within the first minutes after onset of the disease [50], as well as the infiltration of immune cells, which plays a crucial role for the severity and prognosis of pancreatitis [51,52]. Neutrophil granulocytes and monocytes/macrophages, which represent the cells of the innate immune, are the majority of infiltrating cells. One main function of NFκB is the transcriptional regulation of the immune response. It plays a crucial role for the activation of leukocytes and is a central mediator of the innate and adaptive immune system [53,54].

### 6.1. Gallstones

The biliary etiology of pancreatitis has a female predominance, which can be explained by cross-sectional studies showing that 50% of women and only 15% of men have cholelithiasis [55,56]. According to reports by the European Association for the Study of the Liver published in 2016, gallstones in both the gallbladder and the bile duct increase the risk of pancreatitis. Three quarters of patients with cholecystolithiasis are asymptomatic, while 8% of those with gallstones eventually develop AP [57].

Often the first symptom of biliary stones is AP, though most patients recover completely after a mild edematous pancreatitis episode. Unfortunately, 15–30% develop severe necrotizing pancreatitis, necessitating intensive care and multidisciplinary treatment strategies [6]. Acute biliary pancreatitis is triggered by gallstone migration, leading to obstruction of the bile duct or pancreatic duct, or both. The most common location of obstruction is the terminal common bile–pancreatic duct. Most researchers agree that bile–pancreatic duct obstruction and pancreatic hyperstimulation are the main causal factors in acute biliary pancreatitis [49].

Two predominant causal hypotheses, gallstone migration and common pathway [49], agree that bile–pancreatic duct obstruction is the most crucial factor for acute biliary pancreatitis. Duct obstruction increases pancreatic duct pressure, activates bile and trypsin reflux, and causes trypsin activation, pancreatic auto-digestion, and local inflammation. However, such an obstruction does not induce the kind of morphological changes typical for AP, suggesting that other factors must be involved [58]. It is hypothesized, without supportive evidence, that pancreatic acinar hyperstimulation causes and aggravates AP in the presence of duct obstruction [45].

### 6.2. Alcohol Abuse

The second most common cause of AP is alcohol abuse [6,49]. It has been established that excessive alcohol consumption can initiate an episode of AP and increase susceptibility to CP. However, although epidemiological data support the connection between alcohol and pancreatitis, only a minority of alcoholics ever experience AP or CP. This suggests that alcohol consumption is rarely the only factor precipitating pancreatitis. Alcohol sensitizes the pancreas to cofactors such as infectious agents, tobacco smoke, and a high fat diet. Interestingly, tobacco smoke is an independent and probably a stronger risk factor than alcohol consumption in the etiology of CP [6] (Weiss et al., 2019).

According to Gao et al. [22], the development of pancreatitis is influenced by environmental and genetic factors, and alcoholic pancreatitis could be favored by unsuccessful inhibition of trypsin activation or unsuccessful removal of active trypsin from the pancreatic ducts. Thanks to the expression of relevant genes, including aldehyde dehydrogenase (ALDH), alcohol dehydrogenase (ADH), cytochrome P450 (CYP2E1) and catalase, the pancreas, like the liver, can metabolize alcohol by oxidative and non-oxidative pathways. The oxidative pathway generates reactive oxygen species and acetaldehyde; fatty ester ethyl esters, which can injure the pancreas, are formed through the non-oxidative pathway. Ethanol and its metabolites disturb intracellular homeostasis in the pancreatic acini, for instance leading to endoplasmic reticulum stress, impaired autophagy, or activation of lysosomal and pancreatic digestive enzymes [6].

### 6.3. Hypertriglyceridemia

Ten percent of AP cases are associated with an elevated triglyceride level [6]. Most patients with hypertriglyceridemia remain asymptomatic. It is assumed that elevated serum levels of triglycerides and chylomicrons increase blood viscosity, causing local ischemia of the pancreatic tissue. Tissue ischemia changes metabolism from aerobic to anaerobic; cells produce ATP through anaerobic glycolysis, the final product of which is l-lactate. Local ischemia promotes an increase in l-lactate concentration and causes acidosis, which enhances the toxicity of free fatty acids and causes the auto-activation of trypsinogen. When combined with other risk factors such as alcohol, drugs, or tobacco, ischemia associated with hypertriglyceridemia can cause AP.

An idiosyncratic risk of recurrent episodes of AP is observed in patients with familial lipoprotein lipase deficiency [14]. Owing to the increased levels of cholesterol and triglycerides caused by hormones, pregnant women also have an inherent risk of hypertriglyceridemia-induced AP [59].

There are two types of hypertriglyceridemia, primary and secondary, which show specific differences. According to Fredrickson’s classification [60], there are five types of primary hypertriglyceridemia, of which types I, IV, and V are associated with a higher risk of AP. Primary hypertriglyceridemia is caused by inherited autosomal recessive (type I) or autosomal dominant (types IV and V) mutations. Type I is characterized by increased chylomicrons, type IV is associated with high very-low-density lipoproteins (VLDLs), while in type V there are increased concentrations of both chylomicrons and VLDL. Secondary hypertriglyceridemia is associated with modifiable and environmental factors: pregnancy, obesity, inappropriately controlled diabetes, acute and chronic alcohol abuse, or medications.

According to the Atlanta classification system [6], a serum triglyceride level exceeding 1000 mg/dL is the basis for diagnosing hypertriglyceridemia-induced AP. A fifth of all patients meeting this criterion are at increased risk of developing at least one attack of AP.

### 6.4. Endoscopic Retrograde Cholangiopancreatography (ERCP)

An iatrogenic cause of acute pancreatitis is a complication of ERCP, termed post-ERCP acute pancreatitis. Post-ERCP acute pancreatitis is the most common serious adverse event attributed to the procedure. It is defined as pancreatitis with the presence of new or worsened abdominal pain, amylase at least three times normal at more than 24 h after the procedure, requiring admission or prolongation of planned admission to 2–3 days [61,62]. The relevant factors can be patient-related, operator-related, or procedure-related [6,49]. After the procedure, 35–70% of patients exhibit asymptomatic hyperamylasemia. ERCP is associated with a higher risk of inducing AP when the procedure is used to treat a dysfunctional sphincter of Oddi than to remove gallstones from the bile ducts [49]. Patient-related factors are gender, age, Oddi sphincter anomalies or pancreatic anomalies (e.g., pancreas divisium), preexisting pancreatitis, and biliary pancreatitis, an indication for ERCP [6]. Other risk factors for post-ERCP AP include the number of attempts to cannulate a papilla and poor emptying of the pancreatic duct after opacification [6]. Improper canulation of the greater papilla of the duodenum can cause swelling, sphincter contraction, and obstruction of the pancreatic ducts [63]. The physicochemical properties of the contrast medium such as osmolarity, pH, and composition can contribute to hydrostatic and chemical injury to the pancreas. Additionally, the administration of contrast, which entails an increase in pressure, can induce the activation of pancreatic digestive enzymes, which then cause pancreatic autodigestion and local inflammation [64]. In order to prevent post-ERCP AP in high-risk patients, a temporary pancreatic stent is being introduced [6].

### 6.5. Pancreas Divisium

A common congenital anatomical variant of the pancreatic ducts is a lack of connection between the ventral and dorsal ductal systems, called pancreas divisium. This variation can cause inadequate patency or stenosis in the minor papilla, which leads to the prevention of drainage of pancreatic secretions and, consequently, increases the intraductal pressure. However, whether pancreas divisum is associated with AP is controversial [49].

### 6.6. Intraduct Papillary Mucinous Tumor

An intraduct papillary mucinous tumor can also cause AP. The tumor, or mucus produced by the cancer cells, blocks the pancreatic duct or its side branches, or both. A consequence of pancreatic duct obstruction resulting from mucus deposits and pancreatic hyperstimulation is an increase in pressure in the pancreatic ducts. Thus, pancreatic tumors that produce mucus can cause AP by the same mechanism that underpins acute biliary pancreatitis [4].

### 6.7. Autoimmune Pancreatitis

A unique form of chronic pancreatitis is called autoimmune pancreatitis (AIP), in which autoimmunity against unidentified auto antigens is responsible for the chronic fibro-inflammatory responses in the pancreas [65,66]. AIP is classified into type 1 AIP and type 2 AIP on the basis of clinical features, pathological findings, and IgG4 antibody (Ab) responses [67]. In general, it is accepted that type 1 AIP is a pancreatic manifestation of the systemic IgG4-related disease (IgG4-RD). IgG4-RD is a newly defined disease characterized by elevated serum levels of IgG4 Ab and involvement of multiple organs. Predominant pathological feature of IgG4-RD, as well as type 1 AIP, is a massive infiltration of IgG4-expressing plasmacytes into the affected organs, accompanied by storiform fibrosis [68,69,70]. The presence of neutrophils, but not IgG4-expressing plasmacytes, is a pathological characteristic of type 2 AIP [71,72].

It is worth mentioning that Shiokawa et al. [73] were the first to show that the incidence of cancer after the diagnosis of AIP is significantly higher in patients with AIP than in a sex-, age-, and observation period-matched standard population in a multicenter, retrospective cohort study. Yamamoto et al. [74] also showed that the incidence of cancer after the diagnosis of IgG4-RD is markedly higher in patients with IgG4-RD than in a sex-, age-, and observation period-matched standard population. Several case reports showed that the presence of AIP may be more closely associated with the risk of cancers of extra-pancreatic organs, such as the stomach, lung, and prostate, rather than pancreatic cancer. Therefore, it is unlikely that the presence the presence of AIP and/or IgG4-RD promote the development of pancreatic cancer through inflammation-associated carcinogenesis [73,75,76,77], in contrast to chronic pancreatitis, which is one of the strongest risk factors for the development of pancreatic cancer [78]. The large number of AIP patients in whom cancers are detected at or within one year of the AIP diagnosis strongly suggests, according to a concept proposed by Shiokawa et al.—that AIP and/or IgG4-RD sometimes arise as a paraneoplastic syndrome [73].

### 6.8. Genetic Risk

Genetic risk for RAP and CP is similar, whereas, due to the absence of adequate follow-up that can exclude RAP and CP cases, genetic studies in AP are difficult to interpret. As mentioned before, AP, RAP, and CP form a disease continuum [6,79]. Genetic risk factors, as well as chronic alcohol consumption, often lead to the progression of a sentry attack of AP to RAP and eventually to CP. Proteins highly expressed in the pancreas, such as digestive proteases and a trypsin inhibitor, are products of the majority of the pancreatitis risk genes. The various mutations and other genetic alterations, concerning aforementioned pancreatitis risk genes, are classified into pathological pathways responsible for pancreatitis onset and progression, namely the trypsin-dependent, misfolding-dependent, and ductal pathways [80].

Digestive proteases, secreted in inactive precursor forms by pancreatic acinar cells, are flushed from the ductal system in a sodium bicarbonate-rich fluid. The precursor of trypsin-trypsinogen becomes activated by the serine protease enteropeptidase in the duodenum. Additionally, trypsinogen can be activated in the process called autoactivation, in which it is activated by trypsin [81]. Autoactivation or catalyzation by the lysosomal cysteine protease cathepsin B may lead to premature, intra-pancreatic activation of trypsinogen. Protective mechanisms that avert trypsinogen activation in the pancreas comprise trypsin inhibition by the serine protease inhibitor Kazal type 1 (SPINK1) and trypsinogen degradation by chymotrypsin C (CTRC) and cathepsin L [82,83,84]. As mentioned before, the main function of CTRC is to promote trypsinogen degradation, however it also enhances trypsinogen activation by processing the trypsinogen activation peptide to a shorter form, which is more sensitive to trypsin-mediated activation [85,86]. Trypsinogen activation may be stimulated to a pathological extent due to specific trypsinogen mutations that hijack the aforementioned mechanism.

According to the human genetic studies, trypsinogen autoactivation and CTRC dependent trypsinogen degradation are the key mechanisms determining intrapancreatic trypsin activity [80]. Mutations in specific genes have been established, such as PPSS1, SPINK1 and CTRC mutations. Mutations in human cationic trypsinogen cause autosomal dominant hereditary pancreatitis (HP) with incomplete penetrance or act as risk factors in sporadic CP [84]. Additionally, PRSS1 mutations stimulate activation of cationic trypsinogen by reducing CTRC-dependent trypsinogen degradation, increasing CTRC-mediated processing of the activation peptide, or directly stimulating autoactivation. Furthermore, the association between the most common p.N34S SPINK1 variant and CP was proved, making the p.N34S the clinically most SIGNIFICANT risk factor for CP [84]. Moreover, the specific heterozygous mutations in CTRC gene, discovered in patients with nonalcoholic CP by the use of direct DNA sequencing, increased CP risk by 5-fold on average [87]. The mutations cause loss of CTRC function by various mechanisms, which include defective secretion due to misfolding, catalytic deficiency, resistance to trypsin-mediated activation, or increased degradation by trypsin [88]. However, the protective mutations have also been revealed, for instance protective anionic trypsinogen (PRSS2) variant or CTRB1-CTRB2 locus inversion, which reduce CP risk [89,90].

Testing the patients’ DNA may help the clinicians to better understand the pathomechanism of pancreatitis, as well as other diseases, to introduce appropriate therapy. For instance, in the case of RAP, diagnosing a specific genetic CP risk factor allows the introduction of preventive actions. Thorough description of all pathways and connected mutations is too extensive for our paper. We would like to present and thus prove, on the basis of one pathologic pathway, that knowledge in the genetic field is the future of medicine.

### 6.9. Rare Causes

#### 6.9.1. Hypercalcemia

A rare cause of AP is hypercalcemia. The low incidence of AP among patients with chronic hypercalcemia indicates that additional factors are needed for AP to develop [6,91].

#### 6.9.2. Drugs

Cases of drug-induced pancreatitis (DIP) have been reported, although they are rare (0.1–2%) [6]. The most cases of drug-induced pancreatitis (DIP) are mild to moderate in severity. Nevertheless, severe and fatal cases can occur [92]. A retrospective cohort study performed by Sánchez-Aldehuelo et al. [93] included patients with DIP between 2008 and 2018. They concluded that drugs are a rare cause of pancreatitis, and furthermore, DIP mostly occurs within the first month of treatment, is usually mild, and is associated with a low risk of recurrence. Management of DIP includes withdrawal of the suspected active substance and supportive care [92].

Firstly, the diagnosis of DIP requires a diagnosis of AP, which is done on the basis of two of three criteria-typical belt-like abdominal pain, elevated serum lipase level three times above the normal threshold and radiological imaging signs of pancreatitis [19,30,94]. The first two are present in the majority of cases, whereas the latter occurs slightly less frequently. Due to that, in most of the patients, diagnosis of AP can be already established on the basis of abdominal pain and an elevation of pancreatic enzymes [94]. In the second step in diagnosing DIP, more common etiologies, such as gallstone pancreatitis and ethanol-induced acute pancreatitis, have to be ruled out. Therefore, a detailed medical history and the patient’s medications must be established. The history should include previous symptoms and any record of gallstones, ethanol abuse, hypercalcemia, hypertriglyceridemia, and trauma. Additionally, laboratory assays, such as serum amylase, lipase, triglyceride level, calcium level, and liver function tests, should be performed. Moreover, abdominal and endoscopic ultrasounds should be performed to evaluate the condition of bile and pancreatic ducts for gallstones and other obstructive possibilities such as tumors of the pancreas head. ERCP should not be performed after an episode of AP in the absence of imaging or chemical evidence of choledocholithiasis [29,30]. The next step is to discontinue any drugs with the potential to cause pancreatitis or to exchange for a drug of a different class, if possible. Suspicion of DIP increases if the pancreatitis resolves after discontinuation of the drug. Finally, a reliable diagnosis can be established with a rechallenge of the suspected drug that results in the recurrence of pancreatitis symptoms [92,95,96]. Mostly due to delays in diagnosis, cases of DIP are associated with higher morbidity, extended hospital stays, and increased healthcare costs. Therefore, patients presenting with pancreatitis of unknown etiology should be thoroughly questioned regarding drugs that could be connected with DIP [97].

The pathomechanism of DIP is associated with cytotoxic and metabolic effects, sphincter contraction, hypersensitivity reactions, arteriolar thrombosis, and local angioedema. Commonly prescribed drugs associated with AP include angiotensin-converting enzyme (localized angioedema), statins (direct and accumulation toxicity), oral contraceptives or hormone replacement therapy, especially estrogen (hypertriglyceridemia, local arteriolar thrombosis), antiviral therapy (HIV), diuretics, valproic acid, and antidiabetic agents such as GLP-1 mimetic [92,98,99,100]. The best-known medicaments causing DIP are 6-mercaptopurine or azathioprine, isoniazid, loop diuretics, and didanosine [95,97,101].

Other causes of DIP include antibiotics (metronidazole, tetracycline class, erythromycin, ampicillin, ceftriaxone, clarithromycin, trimethoprim-sulfamethoxazole, and nitrofurantoin), immunotherapy (interleukin-2 immunotherapy, programmed cell death protein 1 blockers, anti-cytotoxic T-lymphocyte-associated protein 4 agents), antiacids (2-blockers and proton-pump inhibitors), antidepressants, antiseizure medications, steroids, and non-steroidal anti-inflammatory drugs (NSAIDs) [97]. However, both diclofenac and indomethacin, representatives of NSAIDs, may significantly reduce the risk of post-ERCP acute pancreatitis [102,103]. Furthermore, cases of vitamin-induced acute pancreatitis have been reported involving vitamin D. Cases involved oral vitamin D, where pancreatitis seemed to be associated with the hypercalcemic effect of vitamin D, and a vitamin D-analog (tacolcitol) ointment. Another group that should be mentioned is antineoplastics, both antimetabolite and alkylating [97]. Interestingly, a selective estrogen receptor modulator commonly used for the treatment of estrogen/progesterone receptor positive breast cancer, namely tamoxifen, can also lead to pancreatitis as a side effect. Tamoxifen, like oestrogens, increases the plasma level of triglycerides and liver secretion of Very Low Density Lipoprotein. Furthermore, it inhibits the key enzymes of triglyceride metabolism. However, only few cases of severe tamoxifen induced hypertriglyceridemia and pancreatitis have been reported. Clinicians must be aware of this rare side effect of tamoxifen, as well as in the case of other high-risk drugs. Consequently, while using tamoxifen, baseline and periodic testing of triglyceride level must be done, and care has to be taken, especially in previously diabetic and hypertriglyceridemic females [104].

Many cases go unreported due to the absence of mandatory adverse drug reporting systems, decreasing the ability of clinicians to causally link acute pancreatitis with medications. It is worth introducing similar reporting systems, as well as classification systems or probability assessment scales, in order to facilitate the management of patients. The earliest classification system of drug-induced pancreatitis was developed in 1980 by Trivedi et al. and included three classes [105]. The most recent classification system, developed by Badalov et al. [95], categorized implicated drugs into four classes. Moreover, the adverse drug reaction probability scale by Naranjo et al. [106] can be used to establish the degree of association between a drug and an adverse reaction. The scale determines the probability of an adverse drug reaction on the basis of the cumulative score on 10 questions. Weissman et al. [97] recently proposed a specific drug-induced pancreatitis probability assessment scale, which is modified from the Naranjo scale to be more pancreatitis-specific. It can be helpful in determining the likelihood of DIP based on the aggregate score from a series of 10 questions. It was suggested that the aforementioned scale increases the ability to accurately identify and implicate potential acute pancreatitis-causing drugs.

#### 6.9.3. Viruses

Viral pancreatitis is also described, most often the result of mumps, measles, coxsackie, Epstein–Barr virus, and hepatitis-A virus infections [107]. Recently, Aloysius et al. [108] reported the case of a patient with COVID-19 who presented with AP without other risk factors.

### 6.10. Clinical Aspects

It is substantial to identify the etiology of AP on admission in order to introduce the best and, in the case of some patients, the most specific therapy. The best intervention in biliary AP with cholangitis is early ERCP, in hypertriglyceridemia-induced AP the introduction of lipid-lowering therapy, in the case of obstruction-evoked AP pancreatic stent placement pancreatic duct is performed, while in autoimmune pancreatitis the steroid therapy is implemented [109].

Unfortunately, the IAP/APA guidelines, concerning the management with the patients with AP, inter alia, determining the etiology of the disease, are insufficiently introduced into daily clinical practice. The etiology of AP remains unclear in almost a quarter of all cases, which can be caused by an insufficient diagnostic work-up or other unknown etiological factors. The study by Zádori et al. [109] showed that 5% of the patients left the hospital after the first and second attacks of AP without any imaging at all, whereas 25% of patients had no diagnostic work-up for biliary AP, such as laboratory tests. The greatest insufficiency in etiology screening, amounting to 71–76%, concerned lipid-induced (triglyceride or cholesterol) pancreatitis. In the case of additional diagnostic work-up for all idiopathic AP after index admission, in 91% of the cases there was no search for biliary, anatomic, or cancer etiology by EUS or MRCP, for autoimmune AP in 98% cases, for genetic AP in 99%, or for virus-induced AP after the first attack in 94%.

It is worth mentioning that the cause of about 40% of fatal AP cases is idiopathic, which emphasizes the significance of determining the etiology. Moreover, defining the etiology is crucial for index AP, as well as for preventing recurrent or chronic pancreatitis [109]. It is important to know the latest guidelines and use them in everyday clinical practice.

## 7. Conclusions

This paper is a review of the published studies presenting the most important information about the anatomy, physiology, and pathology of the pancreas. It shows that familiarity with the morphology (anatomy and histology), physiology, and biochemistry of the organ elucidates the pathomechanism of the disease, which has a positive effect on diagnostics and treatment. Overall, the review could provide a useful summary of information about the pancreas and acute pancreatitis for medical students and young doctors, especially for surgeons. Additionally, it could be a useful reminder of details about pancreas morphology, important during surgical procedures, and of risk factors for AP, and provide clinicians with additional information so that they can better diagnose and treat their patient.

## Data Availability

Publicly available datasets were analyzed in this study. This data can be found here: https://pubmed.ncbi.nlm.nih.gov.
